# Author Correction: Temperature-dependent interphase formation and Li^+^ transport in lithium metal batteries

**DOI:** 10.1038/s41467-023-41234-5

**Published:** 2023-09-05

**Authors:** Suting Weng, Xiao Zhang, Gaojing Yang, Simeng Zhang, Bingyun Ma, Qiuyan Liu, Yue Liu, Chengxin Peng, Huixin Chen, Hailong Yu, Xiulin Fan, Tao Cheng, Liquan Chen, Yejing Li, Zhaoxiang Wang, Xuefeng Wang

**Affiliations:** 1 Beijing National Laboratory for Condensed Matter Physics, Institute of Physics, Chinese Academy of Sciences, Beijing, 100190 China; 2 School of Physical Sciences, University of Chinese Academy of Sciences, Beijing, 100049 China; 3 College of Materials Science and Opto-Electronic Technology, University of Chinese Academy of Sciences, Beijing, 100049 China; 4 Institute of Functional Nano and Soft Materials, Soochow University, Suzhou, 215123 China; 5 School of Materials Science and Engineering, University of Shanghai for Science and Technology, Shanghai, 200093 China; 6 Xiamen Institute of Rare Earth Materials, Haixi Institutes, Chinese Academy of Sciences, Xiamen, 361024 China; 7 State Key Laboratory of Silicon Materials, School of Materials Science and Engineering, Zhejiang University, Hangzhou, 310027 China; 8 Tianmu Lake Institute of Advanced Energy Storage Technologies Co. Ltd, Liyang, 213300 China

**Keywords:** Batteries, Batteries, Batteries

Correction to: *Nature Communications* 10.1038/s41467-023-40221-0, published online 25 July 2023

In this article, the author name Suting Weng was incorrectly written as SutingWeng. The original article has been corrected.

