# Supplementary material for: Temperature-dependent interphase formation and Li+ transport in lithium metal batteries
Source: Nat Commun. 2023 Jul 25;14:4474. doi: 10.1038/s41467-023-40221-0 (PMC10368715; doi:10.1038/s41467-023-40221-0)
Supplement: Supplementary file 1 — Supplementary Information [file 41467_2023_40221_MOESM1_ESM.pdf]

Supplementary Information

Temperature-dependent interphase formation and Li^+ transport in lithium metal batteries

SutingWeng[1,2], Xiao Zhang[1,3], Gaojing Yang[1,2], Simeng Zhang[1,3], Bingyun Ma[4], Qiuyan Liu[1,3], Yue Liu[4], Chengxin Peng[5], Huixin Chen[6], Hailong Yu[1], Xiulin Fan[7], Tao Cheng[4], Liquan Chen[1], Yejing Li[1]*, Zhaoxiang Wang[1,2,3]* & Xuefeng Wang[1,2,3,8]*

1 Beijing National Laboratory for Condensed Matter Physics, Institute of Physics, Chinese Academy of Sciences, Beijing 100190, China

2 School of Physical Sciences, University of Chinese Academy of Sciences, Beijing 100049, China

3 College of Materials Science and Opto-Electronic Technology, University of Chinese Academy of Sciences, Beijing 100049, China

4 Institute of Functional Nano and Soft Materials, Soochow University, Suzhou 215123, China

5 School of Materials Science and Engineering, University of Shanghai for Science and Technology, Shanghai 200093, China

6 Xiamen Institute of Rare Earth Materials, Haixi Institutes, Chinese Academy of Sciences, Xiamen 361024, China

7 State Key Laboratory of Silicon Materials, School of Materials Science and Engineering, Zhejiang University, Hangzhou 310027, China

8 Tianmu Lake Institute of Advanced Energy Storage Technologies Co. Ltd., Liyang 213300, China

*Corresponding authors: wxf@iphy.ac.cn; zxwang@iphy.ac.cn; liyejing26@gmail.com.

Inventory of supplementary information

Supplementary Fig. 1 Electrochemical performance of Li||Cu cells.

Supplementary Fig. 2 Temperature dependent physical state of electrolytes.

Supplementary Fig. 3 Temperature dependent behavior of electrolytes.

Supplementary Fig. 4 Raman spectra of three electrolytes at different temperatures.

Supplementary Table 1 Comparison of electrochemical performance of Li||Cu (or Li||stainless-steel disk) cells at the low temperature.

Supplementary Fig. 5 The lithium deposits distribution after the initial deposition in three electrolytes at −20 °C.

Supplementary Fig. 6 Quantification of the "dead" Li^0.

Supplementary Fig. 7 The electrochemical impedance analysis of the Li||Cu cells.

Supplementary Fig. 8 Temperature-dependent electrochemical impedance analysis of LTO85||LTO65 cells.

Supplementary Fig. 9 Interface resistance of the Li||Cu cells.

Supplementary Fig. 10 The influence of substrates on the desolvation process.

Supplementary Fig. 11 Calculated desolvation energies.

Supplementary Fig. 12 Cryo-TEM images of deposited Li metal using $LiPF_6$–EC/DMC electrolyte.

Supplementary Fig. 13 Cryo-TEM images of deposited Li metal using LiFSI–EC/DMC electrolyte.

Supplementary Fig. 14 Cryo-TEM images of deposited Li metal using LiFSI–MTFA/FEC electrolyte.

Supplementary Fig. 15 Mechanical property of SEI layer formed in LiFSI–MTFA/FEC electrolyte.

Supplementary Fig. 16 Distribution of indirect SEI.

Supplementary Table 2 The elemental atomic ratio of the indirect SEI in Supplementary Fig. 16.

Supplementary Fig. 17 The difference between electrolyte and SEI.

Supplementary Fig. 18 Morphology of Li deposits.

Supplementary Fig. 19 Morphology of Li deposits.

Supplementary Fig. 20 The nanostructure of indirect SEI.

Supplementary Fig. 21 The electrochemical impedance analysis.

Supplementary Fig. 22 Interfacial characterization of Li||Cu cell.

Supplementary Fig. 23 Electrochemical performance in LiFSI–MTFA/FEC electrolyte.

Supplementary Fig. 24 The electrolytes working at high voltage.

Supplementary Fig. 25 Electrochemical performance of Cu||LFP pouch cells with LiFSI–MTFA/FEC electrolyte.

Supplementary Fig. 26 The influence of pre-formed SEI layer.

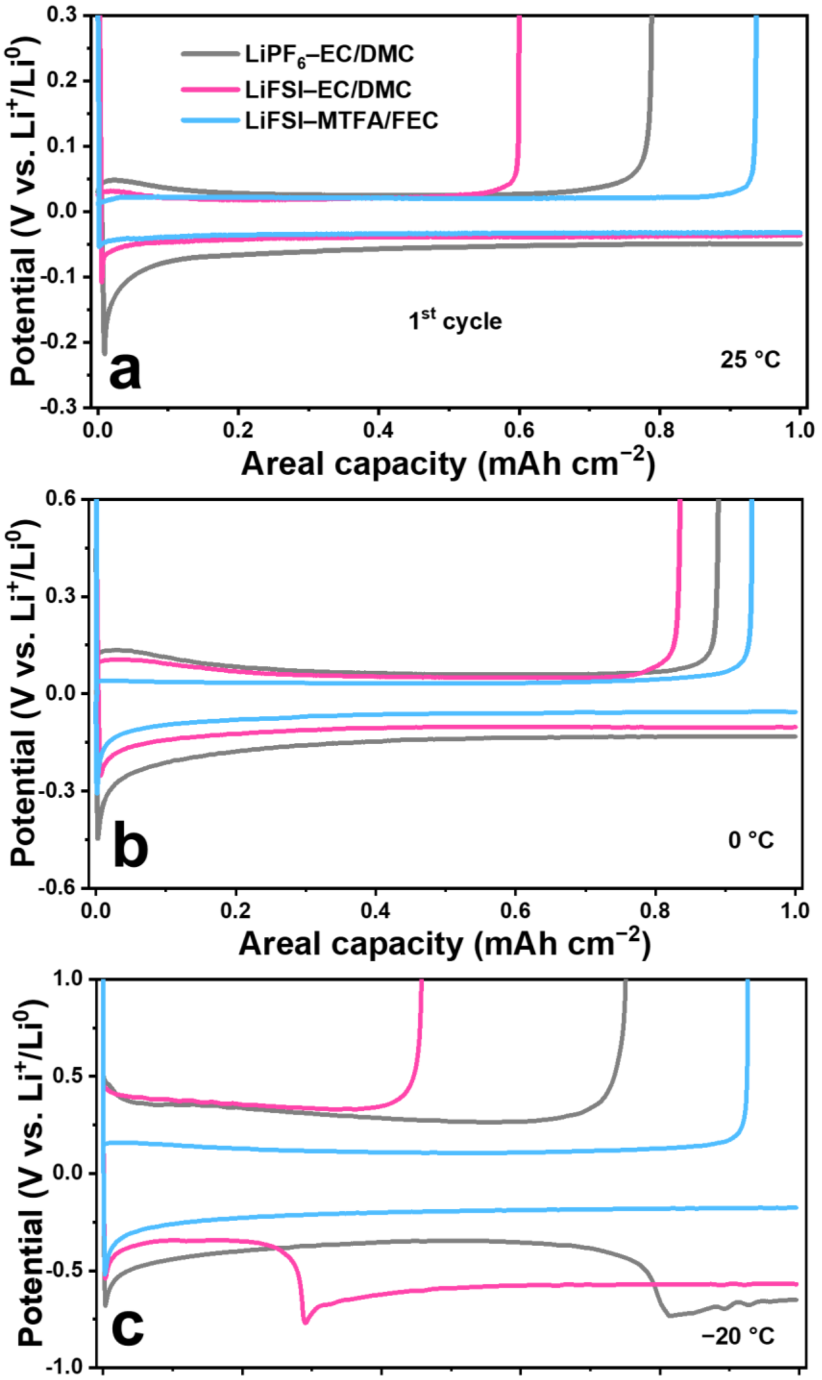

Supplementary Fig. 1 Electrochemical performance of Li||Cu cells. Initial Li plating/stripping potential profiles of Li||Cu cells in three electrolytes under a current density of 0.5 mA cm^{-2} for 1.0 mAh cm^{-2}. **a** 25 °C, **b** 0 °C, and **c** −20 °C.

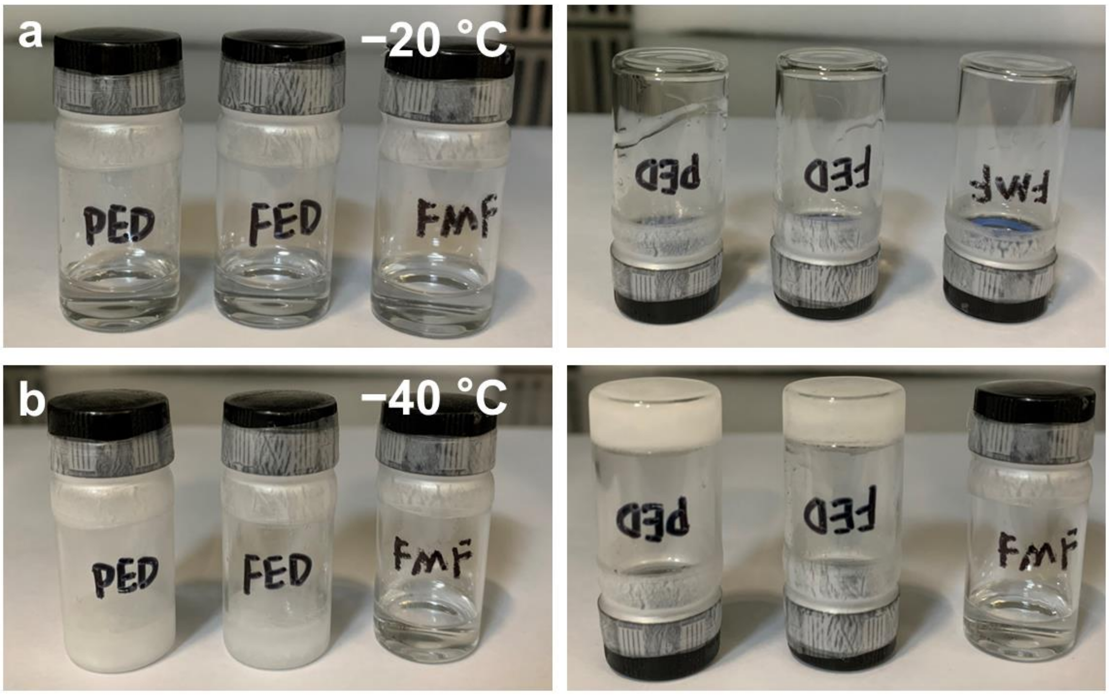

Supplementary Fig. 2 Temperature dependent physical state of electrolytes. Digital photos of three electrolytes **a** −20 °C and **b** −40 °C.

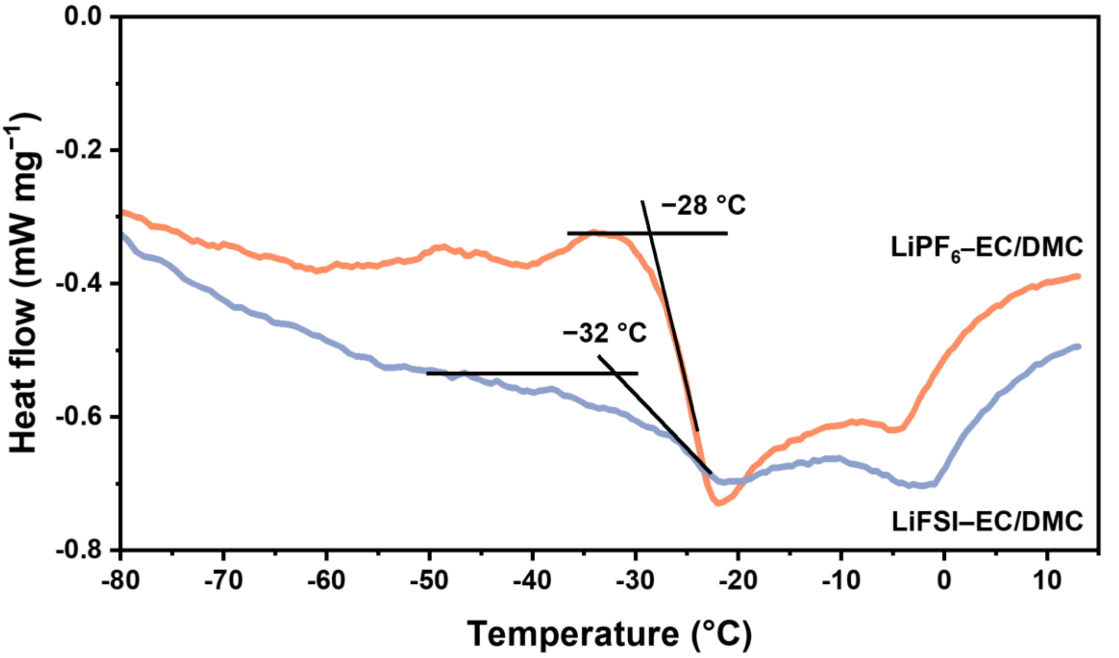

Supplementary Fig. 3 Temperature dependent behavior of electrolytes. Differential scanning calorimetry curves for $LiPF_6$–EC/DMC and LiFSI–EC/DMC electrolytes.

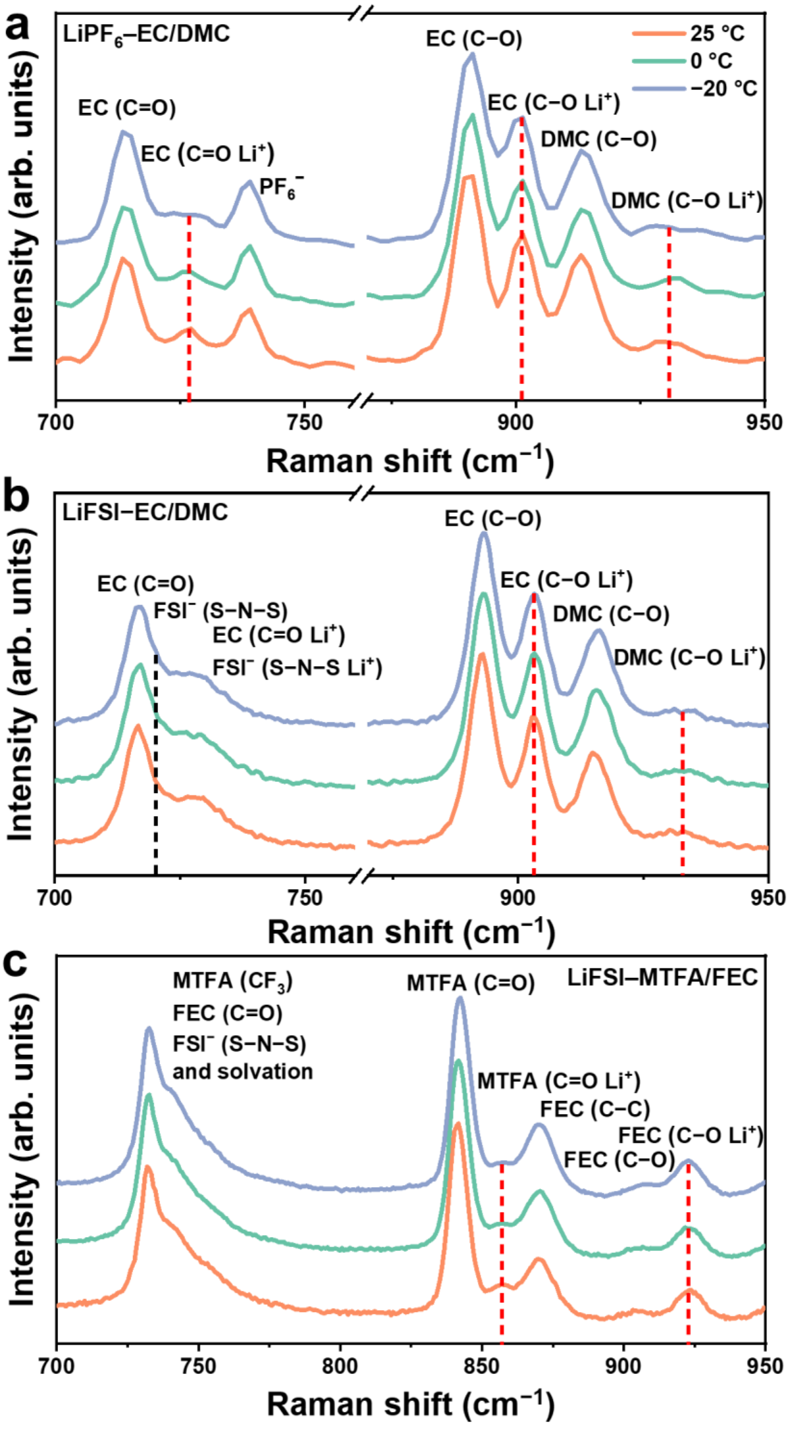

Supplementary Fig. 4 Raman spectra of three electrolytes at different temperatures. **a** $LiPF_6$–EC/DMC, **b** LiFSI–EC/DMC, and **c** LiFSI–MTFA/FEC.

Supplementary Table 1 Comparison of electrochemical performance of Li||Cu (or Li||stainless-steel disk) cells at the low temperature[1-7].

Year	Author	Electrolyte	Current density (mA cm^{-2})	Areal capacity (mAh cm^{-2})	Cycling life		Initial Coulombic efficiency (%)		Lithium deposits morphology	Reference
					−20 °C	−40 °C	−20 °C	−40 °C		
2019	Wang et al. [1]	1.0 M LiTFSI in DOL/DME (1:1, vol) with 1 wt.% $LiNO_3$	1	1	20	-	65.4	-	Roundly shaped (~700 nm at −20 °C) (0.25 mA cm^{-2}–0.15 mAh cm^{-2})	*Nat. Energy* **4**, 664-670 (2019)
2019	Yang et al. [2]	0.3 M LiTFSI + 0.3 M THF in FM:CO_2 (19:1)	0.5	1	-	-	98.6	97.1	-	*Joule* **3**, 1986-2000 (2019)
2019	Thenuwara et al. [3]	1 M LiTFSI in DOL/DME (8:2, vol)	0.5	0.5	50	40	~88	~85	Short rod-like (~500 nm at −20 °C and ~200 nm at −40 °C) (0.5 mA cm^{-2}–4 mAh cm^{-2})	*Nano Lett.* **19**, 8664-8672 (2019)
		1 M LiTFSI +0.2 M $LiNO_3$ in DOL/DME (8:2, vol)	0.5	0.5	50	50	~97	~90	-	
2020	Yang et al. [4]	1.2 M LiTFSI+1 M AN in FM:CO_2 (19:1)	3	3	-	-	97.1 (−30 °C)	94.6 (−60 °C)	Roundly shaped (2 μm at -60 °C) (0.5 mA cm^{-2}–3 mAh cm^{-2})	*Energy Environ. Sci.* **13**, 2209-2219 (2020)
2020	Thenuwara et al. [5]	0.8 M LiTFSI+0.2 M $LiNO_3$ in DOL/DME (8:2, vol)+10 vol% EC	0.5	0.5	50	40	~88	~85	-	*ACS Energy Lett.* **5**, 2411-2420 (2020)
		0.8 M LiTFSI+0.2 M $LiNO_3$ in DOL/DME (8:2, vol)+10 vol% FEC	0.5	0.5	50	50	~97	~90	Roundly shaped (10±2 μm^2 at −40 °C) (0.5 mA cm^{-2}–4 mAh cm^{-2})	
2021	Holoubek et al. [6]	1 M LiFSI DEE	0.5	1	-	-	-	99.0	Massive (~3 μm at −40 °C) (0.5 mA cm^{-2}–5 mAh cm^{-2})	*Nat. Energy* **6**, 303-313 (2021)
2023	Cheng et al. [7]	1 M LiFSI+1 wt.% $LiNO_3$ in MTHF/THF (6:1, vol)	0.5	1	-	-	~96	~85	Roundly shaped (~300 nm at −40 °C) (1 mA cm^{-2}–1 mAh cm^{-2})	*Adv. Funct. Mater.* **33**, 2212349 (2023)
**2023**	**Weng et al.**	**1 M LiFSI in MTFA/FEC (8:2, weight)**	**0.5**	**1**	**100**	**60**	**94.14**	**91.10**	**Dendritic and massive (0.5–2 μm at −20 °C) (0.5 mA cm^{-2}–1 mAh cm^{-2})**	**This work**

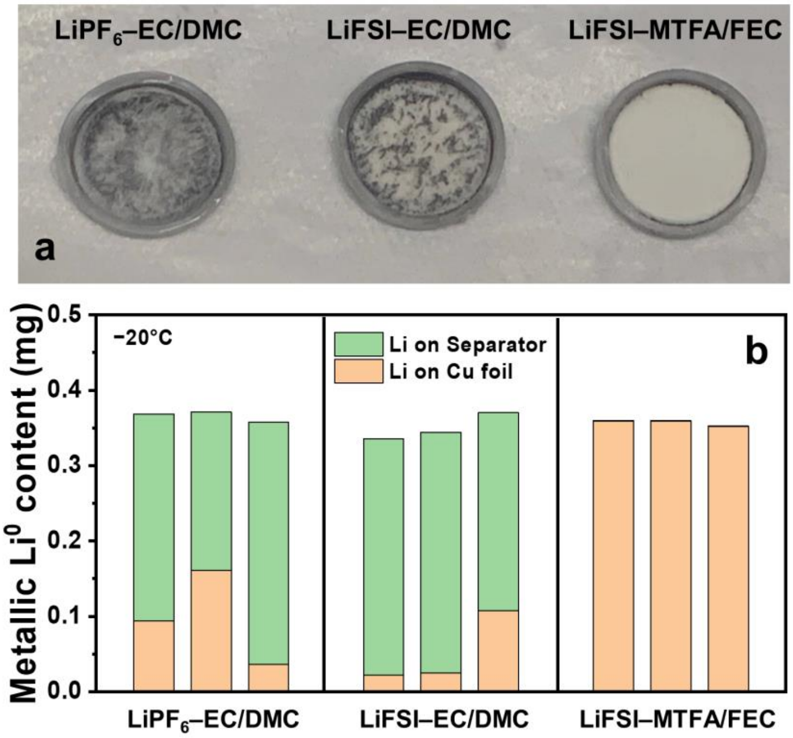

Supplementary Fig. 5 The lithium deposits distribution after the initial deposition in three electrolytes at −20 °C. **a** Digital photographs of separators. **b** the metallic Li^0 content on the separator and Cu foil tested by TGC under a current density of 0.5 mA cm^{-2} for 1.0 mAh cm^{-2}.

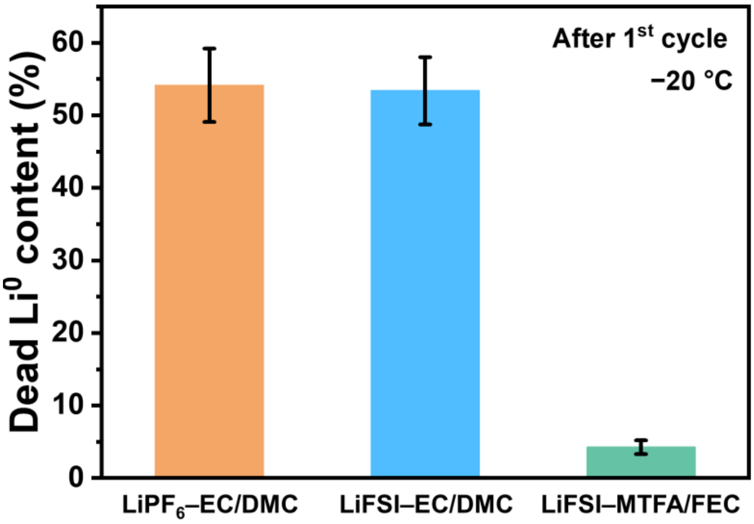

Supplementary Fig. 6 Quantification of the "dead" Li^0. The "dead" Li^0 content of cells in terms of capacity loss with three electrolytes after the initial plating/stripping cycle at −20 °C. The error bars represent the standard deviation of three independent measurements.

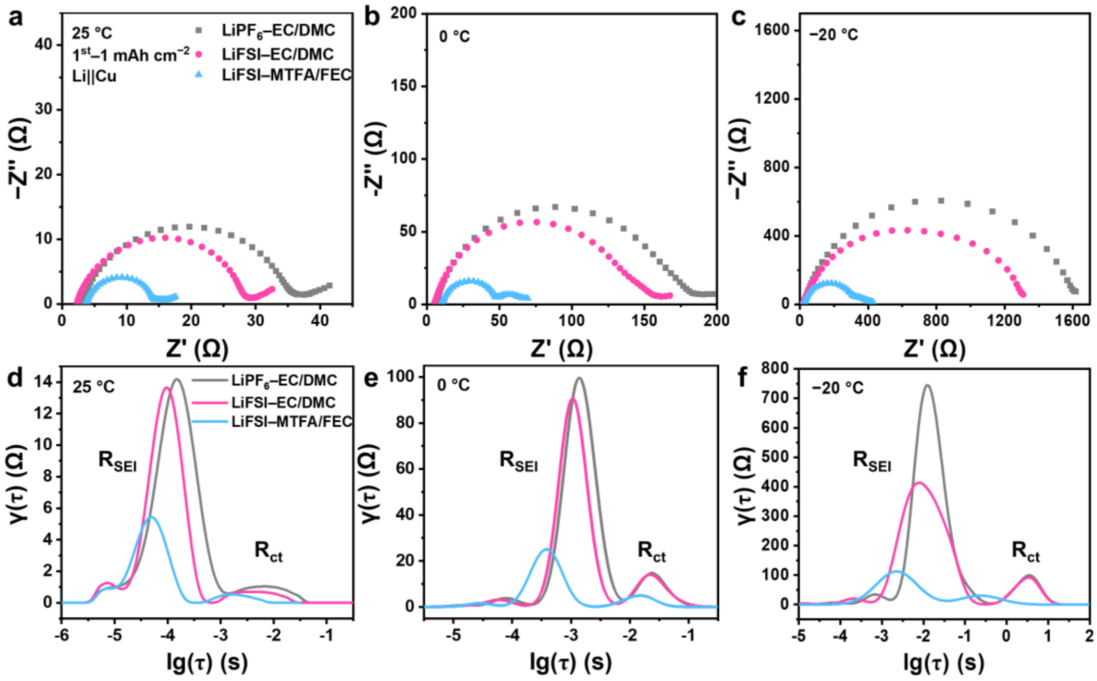

Supplementary Fig. 7 The electrochemical impedance analysis of the Li||Cu cells. **a-c** Electrochemical impedance spectrum (EIS) of the Li||Cu cells after initial deposition (0.5 mA cm^{-2}, 1.0 mAh cm^{-2}) and **d-f** corresponding distribution of relaxation times (DRT) analysis.

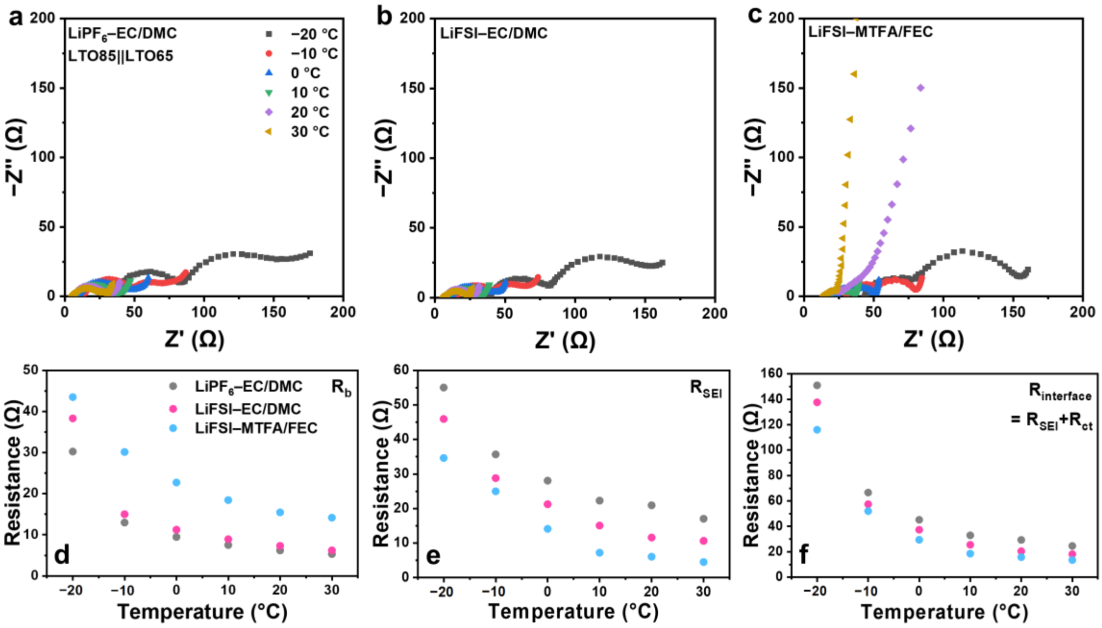

Supplementary Fig. 8 Temperature-dependent electrochemical impedance analysis of LTO85||LTO65 cells. a-c EIS and **d-f** the fitting results of R_b, R_{SEI} and $R_{interface}$.

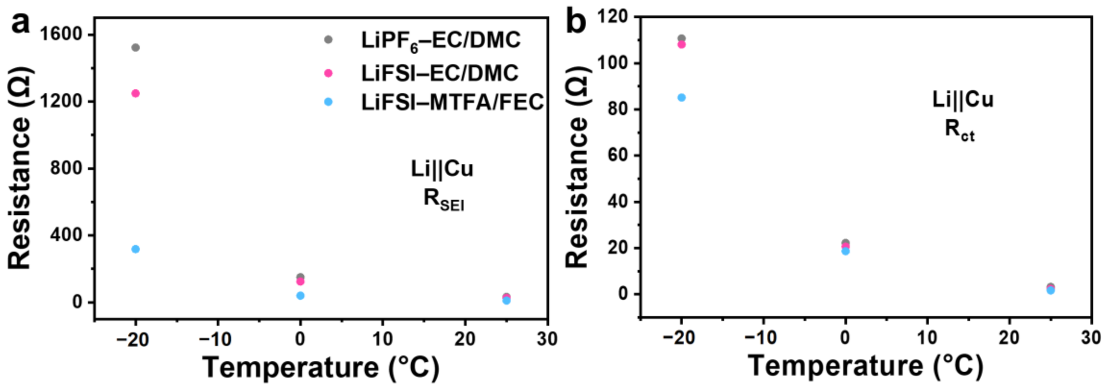

Supplementary Fig. 9 Interface resistance of the Li||Cu cells. The R_{SEI} (**a**) and R_{ct} (**b**) obtained by integrating the natural logarithm ln(τ) of the time constant τ with γ(τ) using RelaxIS software.

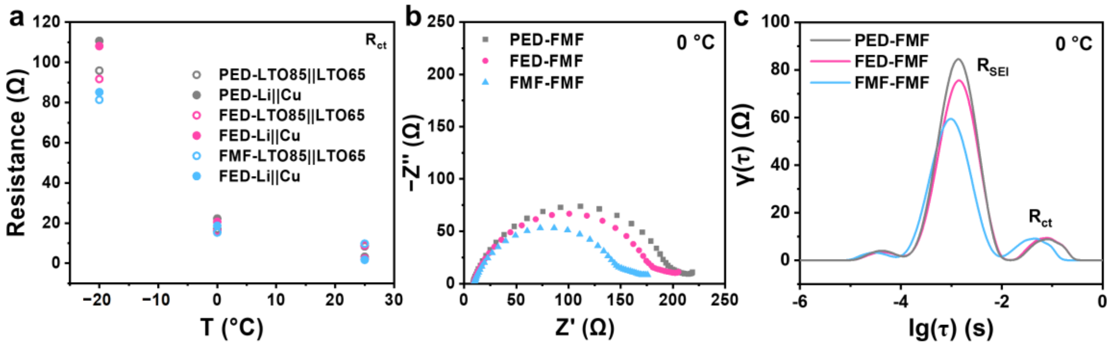

Supplementary Fig. 10 The influence of substrates on the desolvation process. a The R_{ct} values comparison of LTO85||LTO65 cells and Li||Cu cells. The EIS (**b**) and corresponding DRT analysis (**c**) of the Li||Cu cells precycled in one electrolyte (PED, FED, or FMF) for 10 cycles at 0 °C to form SEI layer and then switched to FMF electrolyte, denoted as PED-FMF, FED-FMF, and FMF-FMF, respectively. Similar value of R_{ct} is obtained with different substrates including LTO/Li metal, and Li metal with varied pre-formed SEI suggesting the negligible influence of substrates on the desolvation process when compared with electrolyte.

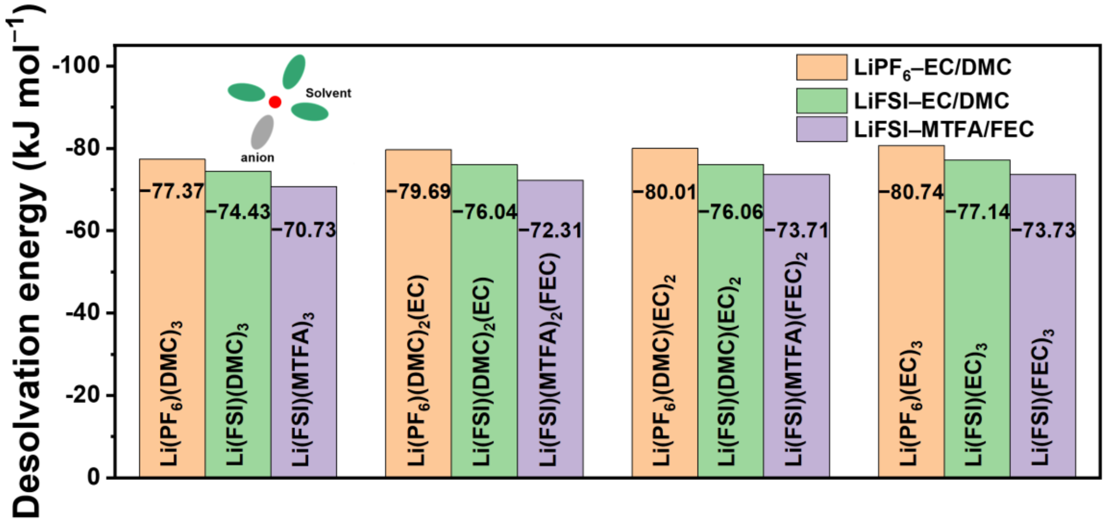

Supplementary Fig. 11 Calculated desolvation energies. Calculated desolvation energies of three electrolytes with different solvation configurations.

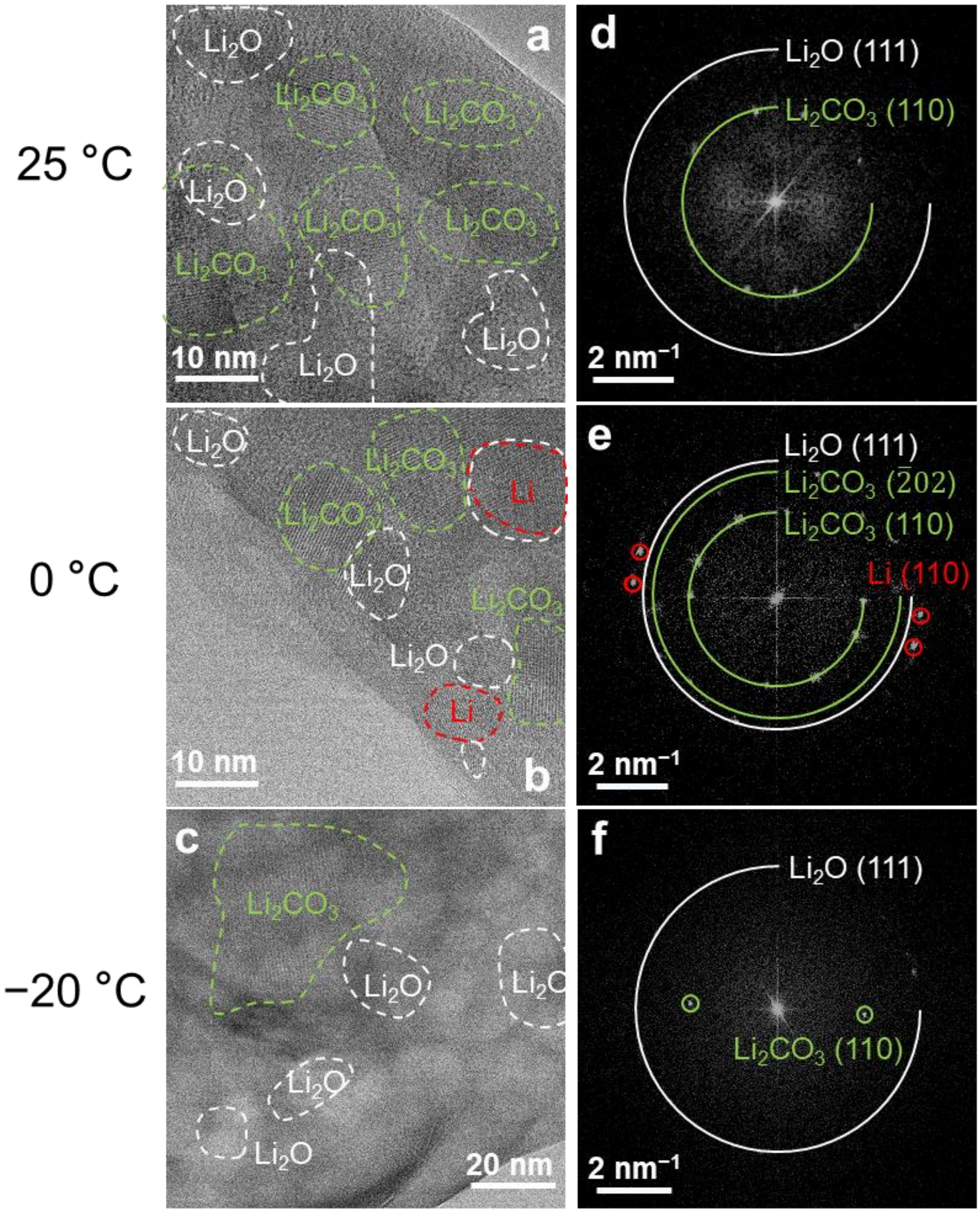

Supplementary Fig. 12 Cryo-TEM images of deposited Li metal using $LiPF_6$–EC/DMC electrolyte. a-c Cryo-HRTEM images and **d-f** corresponding fast Fourier transform (FFT) pattern at different temperatures. (Larger images corresponding to Fig. 5a, d, and g)

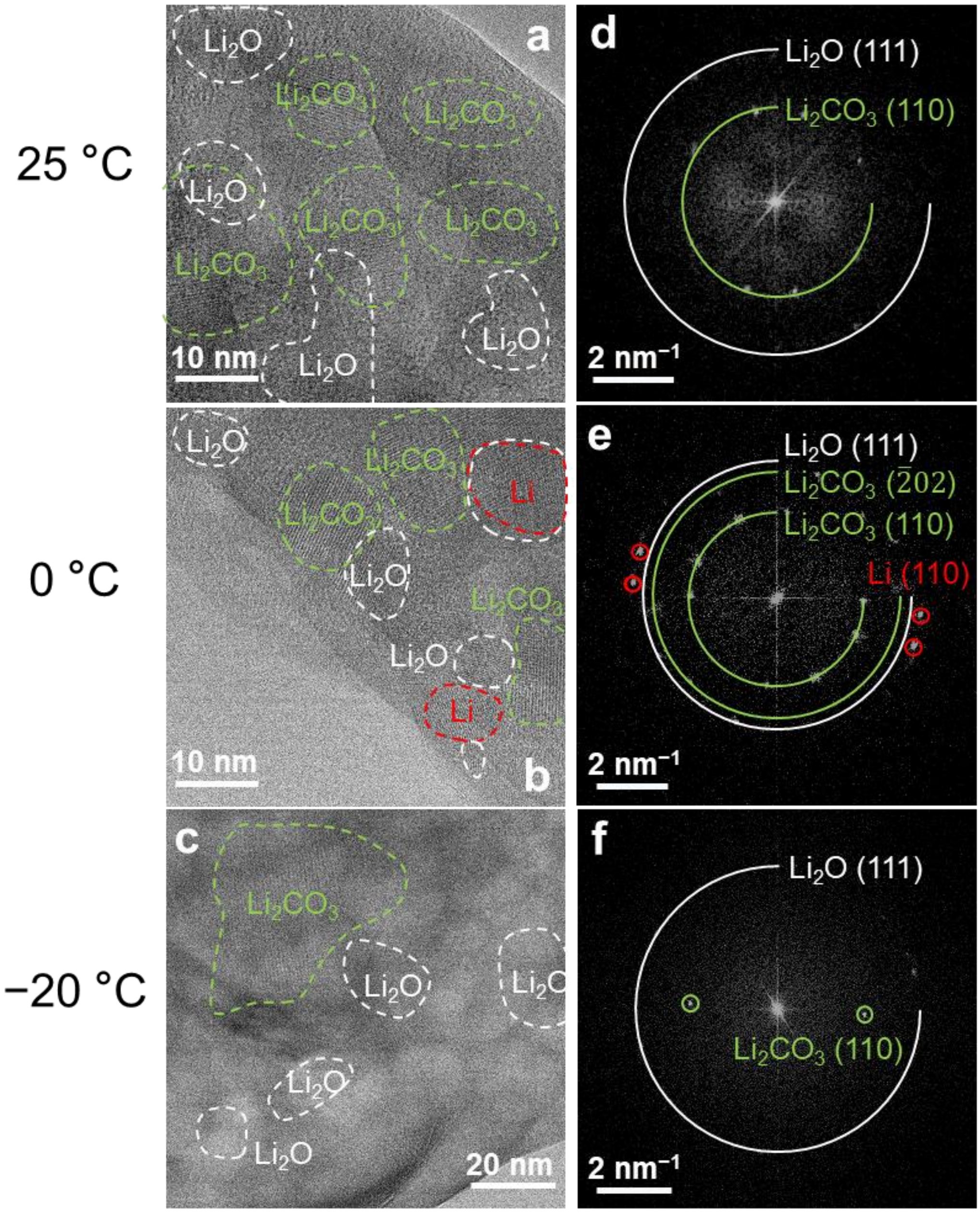

Supplementary Fig. 13 Cryo-TEM images of deposited Li metal using LiFSI–EC/DMC electrolyte. a-c Cryo-HRTEM images and **d-f** corresponding FFT pattern at different temperatures. (Larger images corresponding to Fig. 5b, e, and h)

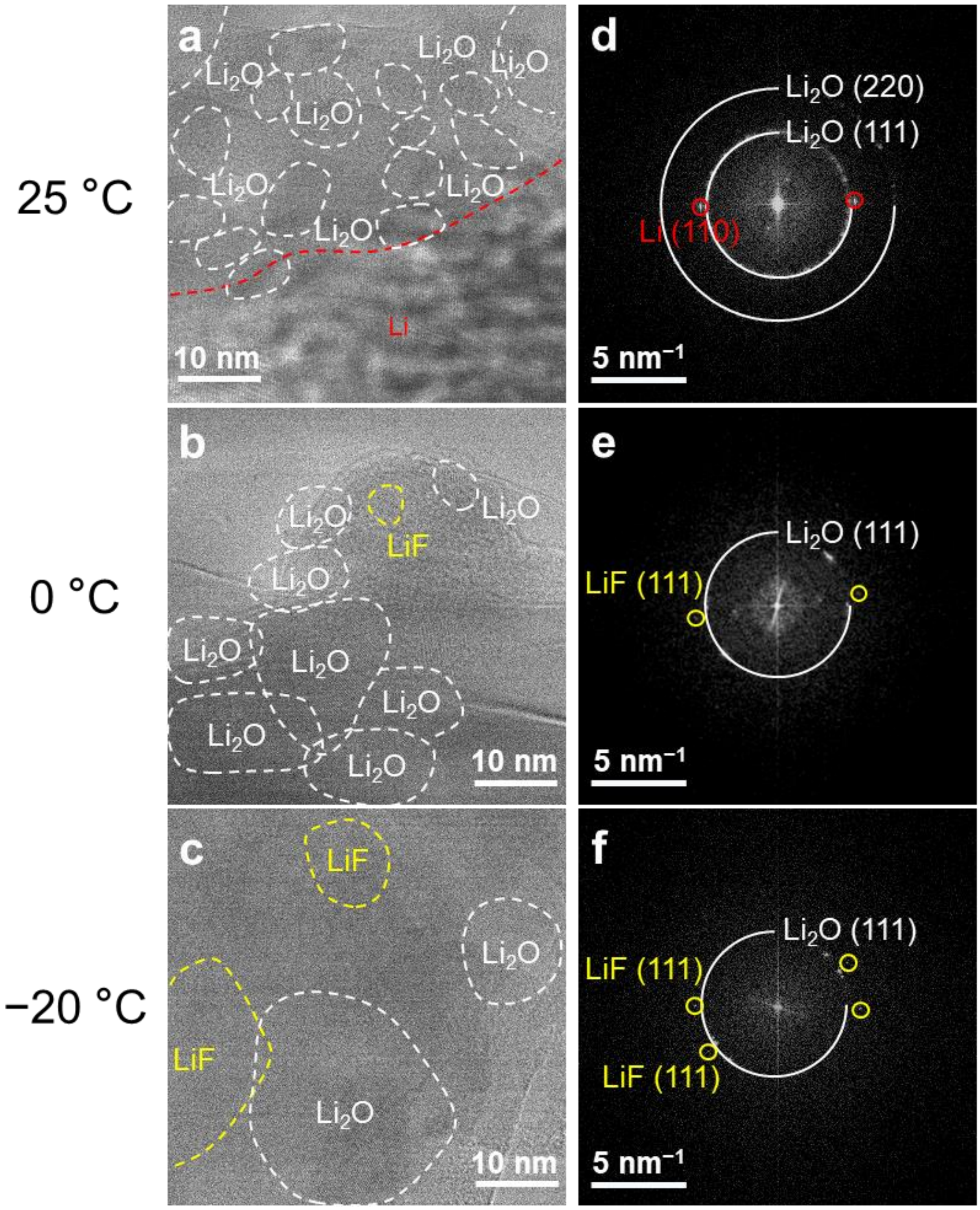

Supplementary Fig. 14 Cryo-TEM images of deposited Li metal using LiFSI–MTFA/FEC electrolyte. a-c Cryo-HRTEM images and **d-f** corresponding FFT pattern at different temperatures. (Larger images corresponding to Fig. 5c, f, and i)

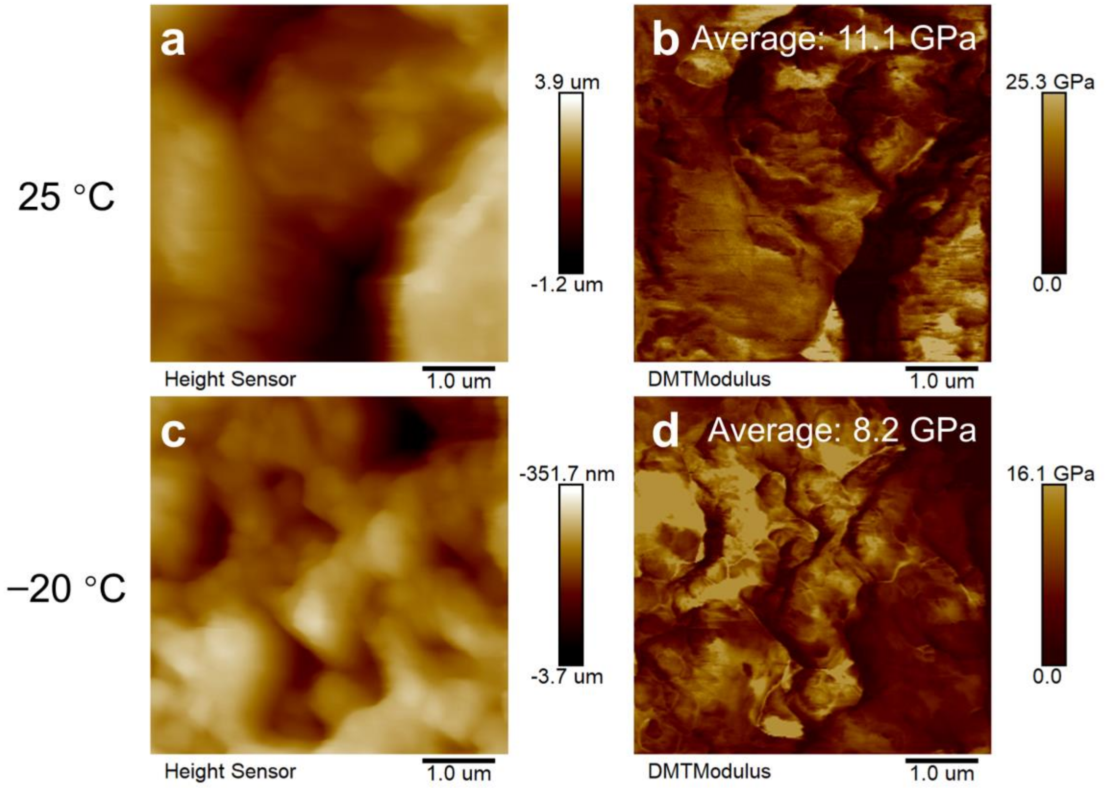

Supplementary Fig. 15 Mechanical property of SEI layer formed in LiFSI–MTFA/FEC electrolyte. a-b 25 °C and **c-d** −20 °C. The FMF-derived SEI layer at 25 °C manifests a significantly higher average Young's modulus (11.1 GPa) than −20 °C (8.2 GPa), representing an enhanced capability in suppressing the dendrite growth.

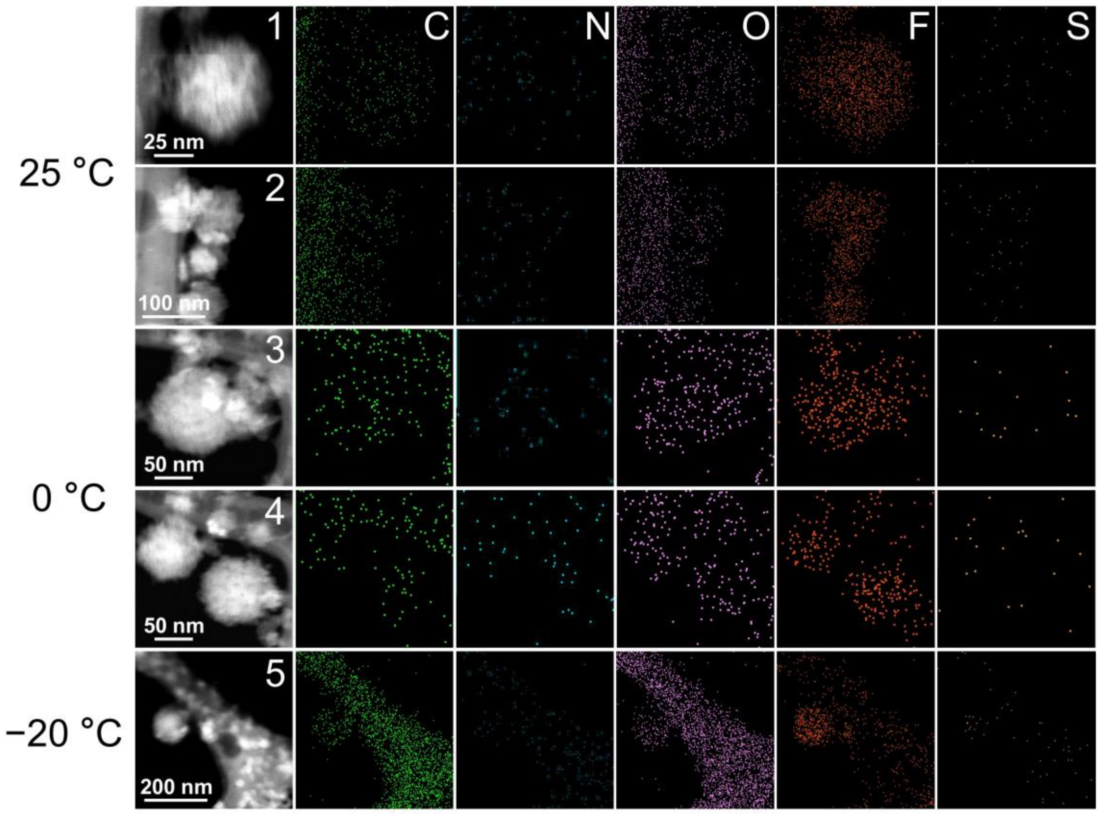

Supplementary Fig. 16 Distribution of indirect SEI. The EDS mapping of the indirect SEI at different temperatures in LiFSI–MTFA/FEC electrolyte.

Supplementary Table 2 The elemental atomic ratio of the indirect SEI in Supplementary Fig. 16.

Element (%)	25 °C		0 °C		−20 °C
	1	2	3	4	5
C	43.44	51.34	47.39	41.32	55.12
N	4.25	4.73	4.28	2.96	6.96
O	9.64	7.90	12.69	15.03	4.29
F	42.28	35.83	35.23	40.11	33.18
S	0.39	0.21	0.42	0.59	0.44

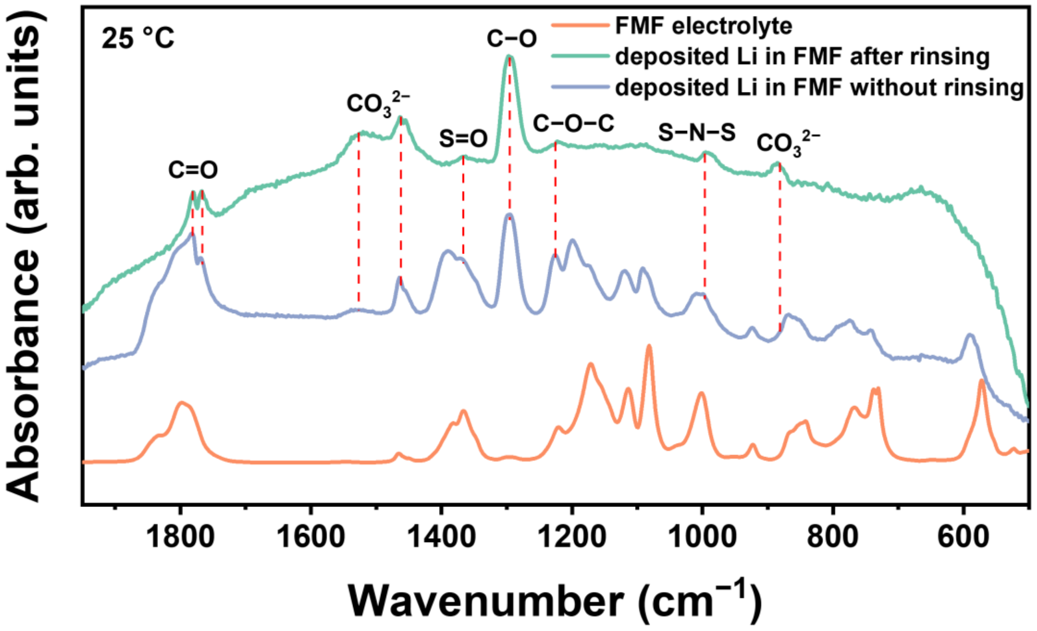

Supplementary Fig. 17 The difference between electrolyte and SEI. FTIR spectra of LiFSI–MTFA/FEC electrolyte and deposited Li after/without rinsing.

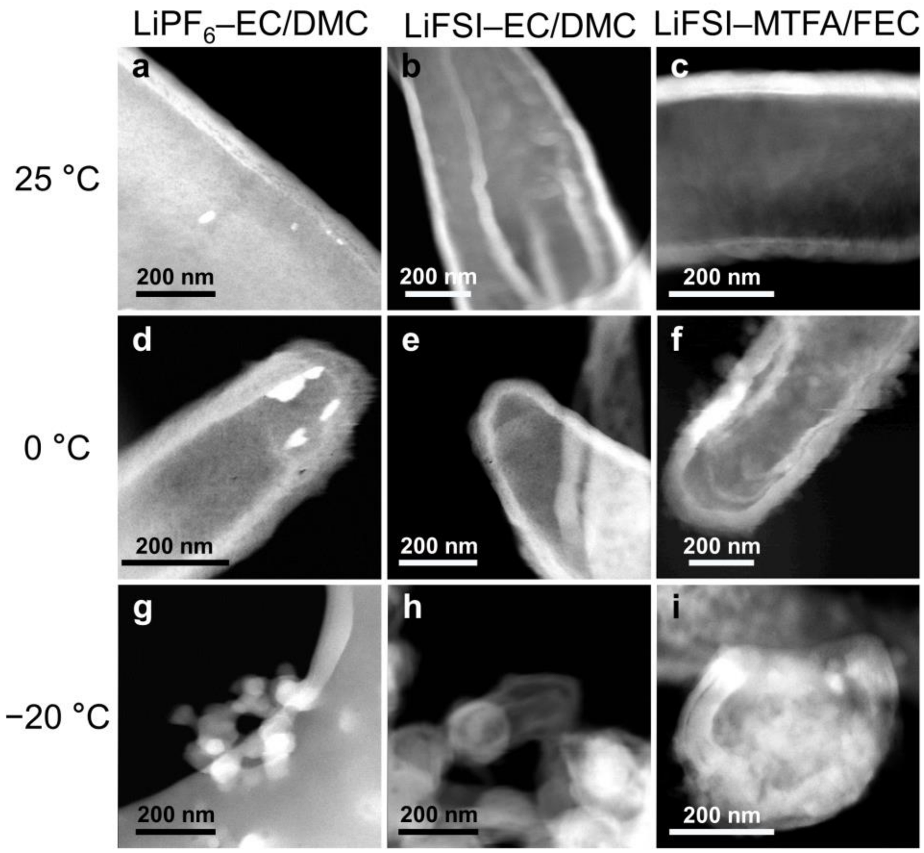

Supplementary Fig. 18 Morphology of Li deposits. Cryo-STEM images of Li deposits using electrolytes of $LiPF_6$-EC/DMC (**a**, **d**, and **g**), LiFSI-EC/DMC (**b**, **e**, and **h**), and LiFSI-MTFA/FEC (**c**, **f**, and **i**) at 25 °C (**a-c**), 0 °C (**d-f**), and −20 °C (**g-i**).

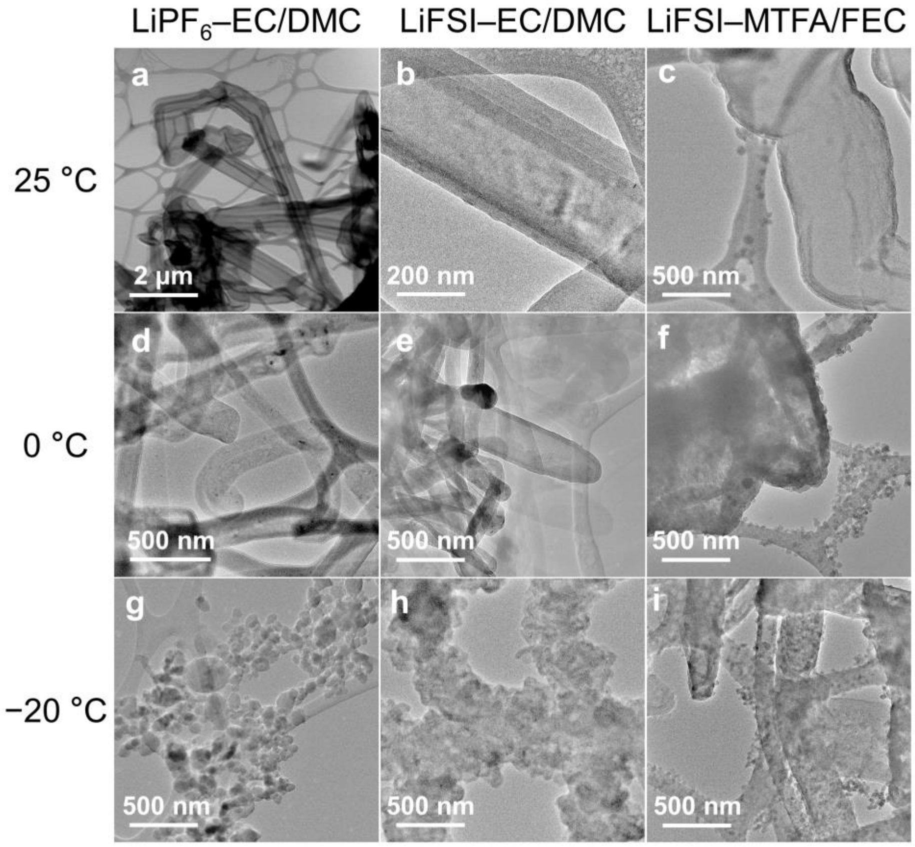

Supplementary Fig. 19 Morphology of Li deposits. Cryo-TEM images of deposited lithium using electrolytes of $LiPF_6$-EC/DMC (**a**, **d**, and **g**), LiFSI-EC/DMC (**b**, **e**, and **h**), and LiFSI-MTFA/FEC (**c**, **f**, and **i**) at 25 °C (**a-c**), 0 °C (**d-f**), and −20 °C (**g-i**).

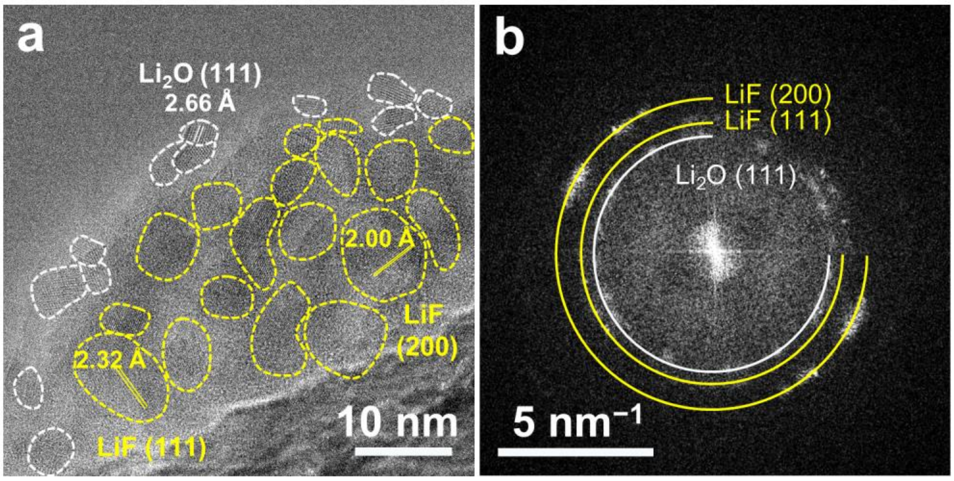

Supplementary Fig. 20 The nanostructure of indirect SEI. a Cryo-HRTEM image and **b** corresponding FFT pattern of indirect SEI in LiFSI–MTFA/FEC electrolyte at 25 °C. (A larger image corresponding to Fig. 6b)

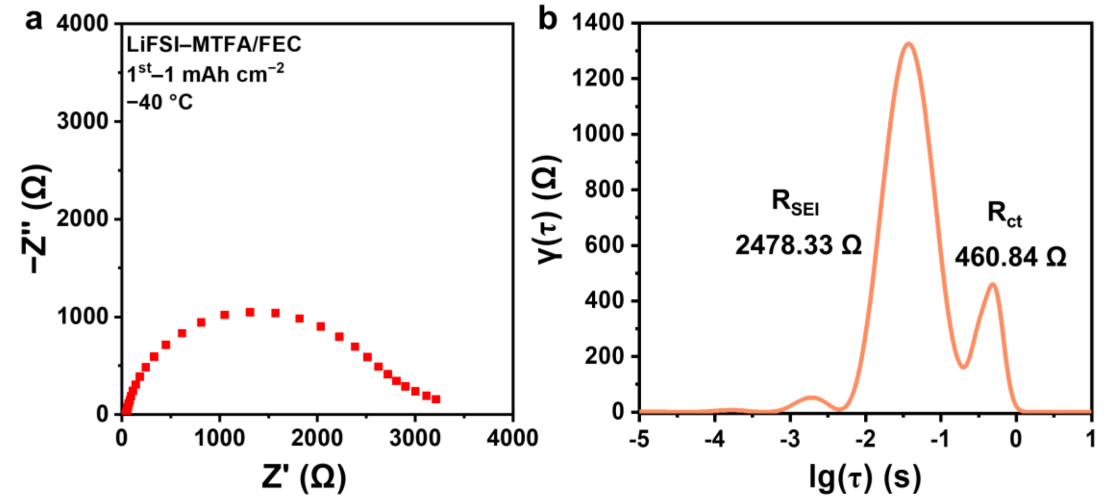

Supplementary Fig. 21 The electrochemical impedance analysis. a EIS of the Li||Cu cells with LiFSI–MTFA/FEC electrolyte at −40 °C after initial deposition (0.5 mA cm^{-2}, 1.0 mAh cm^{-2}) and **b** corresponding DRT analysis.

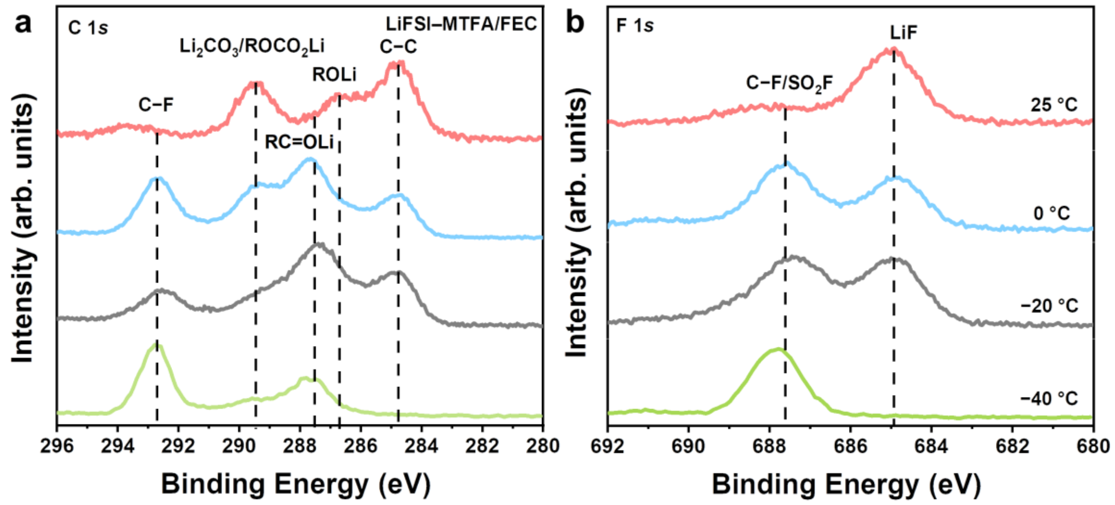

Supplementary Fig. 22 Interfacial characterization of Li||Cu cell. a C 1*s* and **b** F 1*s* XPS spectra of the SEI layer on the deposited Li metal in different electrolytes and temperatures.

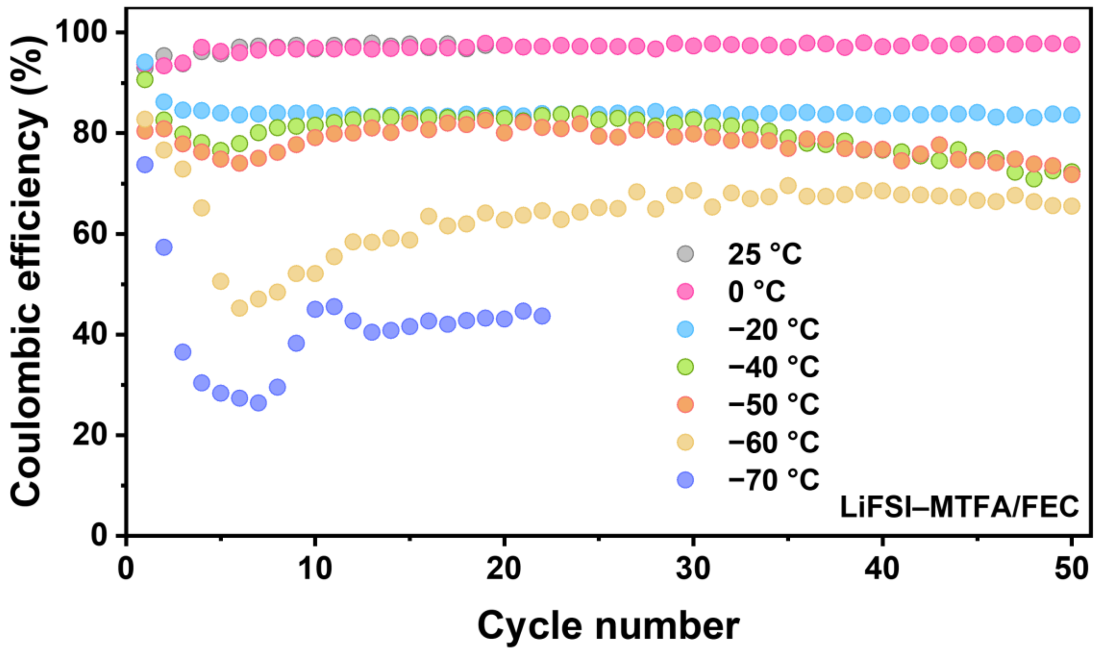

Supplementary Fig. 23 Electrochemical performance in LiFSI–MTFA/FEC electrolyte. Coulombic efficiencies of Li||Cu cells in LiFSI–MTFA/FEC electrolyte under a current density of 0.5 mA cm^{-2} for 1.0 mAh cm^{-2} at 25 – −70 °C.

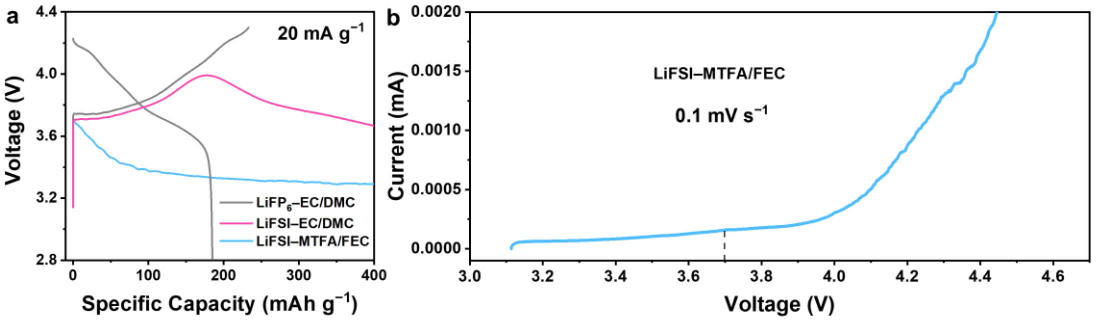

Supplementary Fig. 24 The electrolytes working at high voltage. a The initial charge/discharge profiles of Li||NCM811 cells with three electrolytes with a voltage range of 2.7–4.3 V at 25 °C. **b** The linear sweep voltammetry profile of LiFSI–MTFA/FEC electrolyte on a Ti alloy electrode at a scan rate of 0.1 mV s^{-1}.

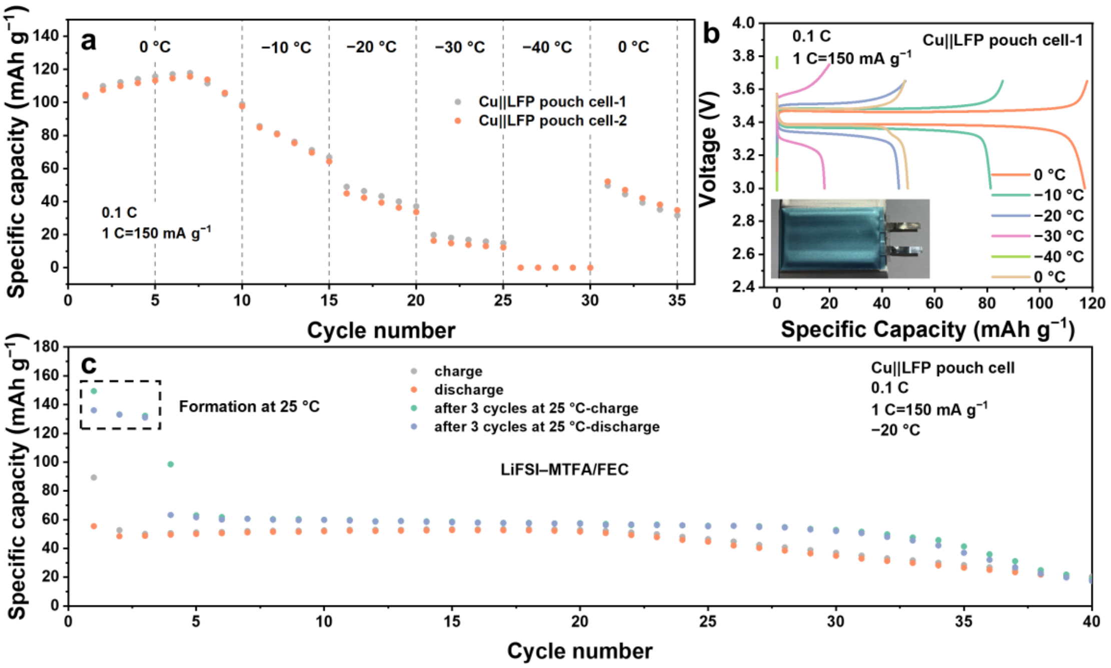

Supplementary Fig. 25 Electrochemical performance of Cu||LFP pouch cells with LiFSI–MTFA/FEC electrolyte. **a** The electrochemical performance and **b** voltage profiles of Cu||LFP pouch cells at different temperatures. **c** The cycling performance of Cu||LFP pouch cells with/without pre-formed SEI layer at 25 °C for 3 cycles in LiFSI–MTFA/FEC electrolyte at −20 °C.

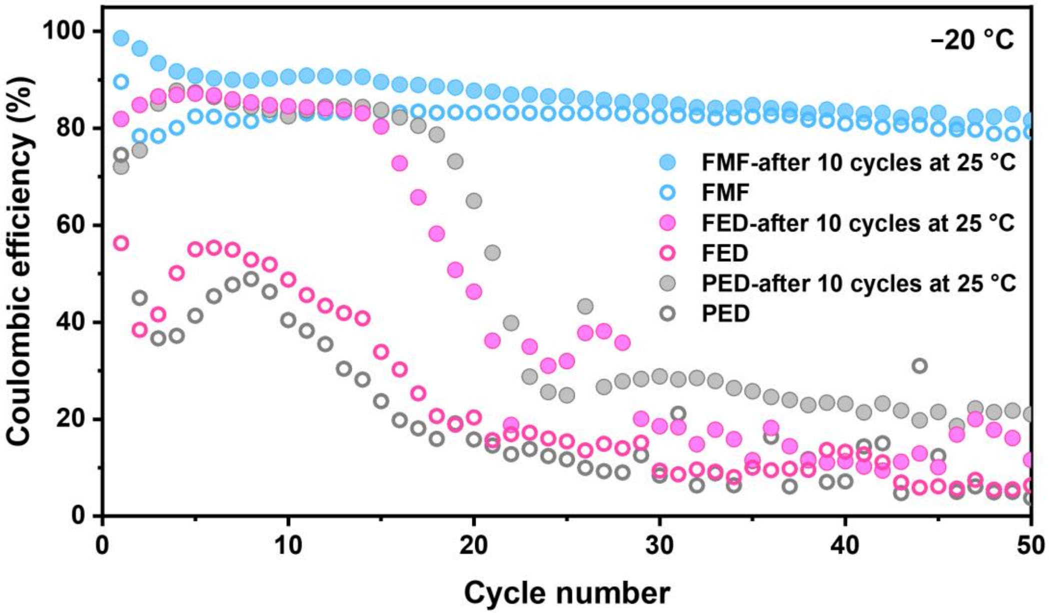

Supplementary Fig. 26 The influence of pre-formed SEI layer. The Coulombic efficiencies of Li||Cu cells with/without pre-formed SEI layer at 25 °C for 10 cycles in three electrolytes under a current density of 0.5 mA cm^{-2} for 1.0 mAh cm^{-2} at −20 °C.

Supplementary References

1. Wang, J. et al. Improving cyclability of Li metal batteries at elevated temperatures and its origin revealed by cryo-electron microscopy. *Nat. Energy* **4**, 664-670 (2019).
2. Yang, Y. et al. High-efficiency lithium-metal anode enabled by liquefied gas electrolytes. *Joule* **3**, 1986-2000 (2019).
3. Thenuwara, A. C., Shetty, P. P. & McDowell, M. T. Distinct nanoscale interphases and morphology of lithium metal electrodes operating at low temperatures. *Nano Lett.* **19**, 8664-8672 (2019).
4. Yang, Y. et al. Liquefied gas electrolytes for wide-temperature lithium metal batteries. *Energy Environ. Sci.* **13**, 2209-2219 (2020).
5. Thenuwara, A. C. et al. Efficient low-temperature cycling of lithium metal anodes by tailoring the solid-electrolyte interphase. *ACS Energy Lett.* **5**, 2411-2420 (2020).
6. Holoubek, J. et al. Tailoring electrolyte solvation for Li metal batteries cycled at ultra-low temperature. *Nat. Energy* **6**, 303-313 (2021).
7. Cheng, L. et al. An ultrafast and stable Li-metal battery cycled at −40 °C. *Adv. Funct. Mater.* **33**, 2212349 (2023).
